# Short-term effects of goat milk yogurt-containing angiotensin-converting enzyme inhibitory peptides and two raisin varieties on subjective appetite, blood pressure and glycaemic responses in healthy adults. Results from a randomised clinical trial

**DOI:** 10.1017/S0007114522002537

**Published:** 2023-07-28

**Authors:** Emilia Papakonstantinou, Eugenia Manolopoulou, Argyris Papamichalopoulos, Chryssi Kounenidaki, Theodora Mitrogeorgou, Marina Georgalaki, Effie Tsakalidou

**Affiliations:** 1 Laboratory of Dietetics and Quality of Life, Department of Food Science and Human Nutrition, School of Food and Nutritional Sciences, Agricultural University of Athens, Greece; 2 Laboratory of Dairy Research, Department of Food Science and Human Nutrition, School of Food and Nutritional Sciences, Agricultural University of Athens, Greece

**Keywords:** Goat milk yogurt, Raisins, Postprandial glycaemia, Glycaemic index, Glycaemic load

## Abstract

Goat milk yogurt (GMY) and raisins are popular foods with a favourable nutrient profile. Our aim was to determine the glycaemic index (GI) and postprandial responses to GMY-containing angiotensin-converting enzyme inhibitory (ACE-I) peptides carrying the RPKHPINHQ isracidin fragment and two Greek raisin varieties in an acute feeding setting. A total of twelve healthy participants (four male and eight female) consumed breakfast study foods containing 25 g available carbohydrate on seven occasions over a 3- to 9-week period: food 1: D-glucose (25 g) served as the control and was consumed on three separate occasions; food 2: GMY (617·28 g); food 3: Corinthian raisins (37·76 g); food 4: Sultana raisins (37·48 g) and food 5: GMY & C (308·64 g GMY and 18·88 g C). Postprandial glucose was measured over a 2 h period for the determination of GI and glycaemic load (GL). Subjective appetite ratings (hunger, fullness and desire to eat) were assessed by visual analogue scales (100 mm) at 0–120 min. Blood pressure (systolic and diastolic; BP) was measured at baseline and 120 min. GMY provided low GI (26), C and S provided high GI/low GL (75/10 and 70/9, respectively) and GMYC provided low GI (47) values on glucose scale compared with D-glucose. Peak blood glucose rise was significantly lower only for GMY and GMYC compared with reference food (D-Glucose), as well as C and S (*P*_for all_ < 0·05). No differences were observed between test foods for fasting glucose, BP and subjective appetite. In conclusion, GMY and GMYC attenuated postprandial glycaemic responses, which may offer advantages to glycaemic control.

## Graphical abstract

**Figure:**
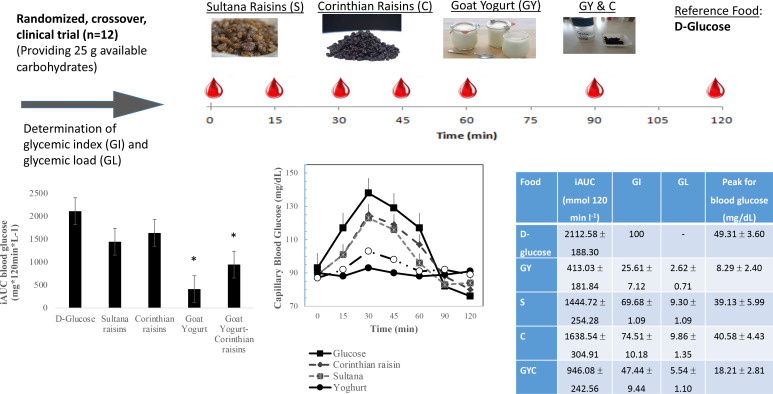

Type 2 diabetes mellitus, obesity and hypertension are amongst the most important public health concerns globally. Consumption of high glycaemic index (GI) foods is associated with increased chronic disease risk^(1,2)^. The GI of foods is a method by which foods can be ranked on the basis of the glycaemic impact^(3)^ in relation to their carbohydrate content^(2)^.

Yogurt consumption has been suggested as a nutritional approach in obesity and Type 2 diabetes mellitus prevention and/or management^(4)^. Yogurt consumption has been shown to ameliorate postprandial hyperglycaemia, lead to better satiety and lower body fat^(5–8)^ possibly due to its high protein and calcium content and its potential probiotic characteristics^(3,8)^. Yogurt is a low to medium GI food with values ranging between 11 and 67^(9)^. Worldwide, the contribution of goat milk to the total milk production remains low (2·6 %), still, however, ranking third after cow (83 %) and buffalo (13 %) milk^(10)^. Interestingly, Greece has a long tradition in small ruminant farming, and among the EU countries, is positioned first in goat breeding (with 3·600·000 goats), which makes almost 30 % of the EU total^(11)^. Moreover, in the past 12 years, the annual goat milk production increased by 12·4 % reaching 562 million litres^(12)^, with the major part being used for cheese making, mainly in combination with sheep milk. In recent years, not only in Greece but around the globe, pasteurised goat milk as well as goat milk yogurt (GMY) have become increasingly popular among consumers. This is largely due to recent findings regarding the nutritional value ascribed to goat milk and its health benefits for humans^(13)^. These are mostly related to higher digestibility and lower allergenicity of goat milk proteins compared with cow milk ones^(14,15)^ and better absorption of fat and minerals^(16)^. Thus, despite technological hurdles regarding GMY production, such as formation of weak consistency gel^(17)^, the development of GMY and in particular enriched with functional ingredients is among the emerging challenges for the dairy sector. Moreover, there is a growing interest in the potential of fermented dairy foods to prevent hypertension through bioactive peptides encrypted within milk proteins that can be released during fermentation with specific lactic acid bacteria or during gastrointestinal digestion. The angiotensin-converting enzyme (ACE) plays a fundamental role in blood pressure (BP), as it converts angiotensin I into angiotensin II, a potent vasoconstrictor. Inhibition of ACE causes a vasodilator response, leading to decreased BP. Our group has identified *in vitro* the presence of three ACE-Inhibitory (ACE-I) peptides carrying the RPKHPINHQ fragment of the so-called isracidin α_s1_-casein peptide in goat milk fermented with either *Lactobacillus delbrueckii* subsp. *bulgaricus* ACA-DC 87 or *Streptococcus thermophilus* ACA-DC 835 strain^(18)^. However, the effects of ACE-I peptides carrying the RPKHPINHQ fragment on BP in healthy humans are inconclusive^(19)^. Moreover, the effects of GMY containing these peptides on postprandial glycaemic responses and appetite when consumed as a preload before an *ad libitum* meal have not been investigated.

Although health benefits of grapes and wine have been studied extensively, dried grapes have received comparatively little attention. Corinthian raisins are small sun-dried vine fruits, produced almost exclusively in Peloponnese, Greece, with the world production corresponding to around 3–5 % of dried vine fruit production^(20)^. They contain neither fat nor cholesterol and have high content of fibres, minerals, vitamins and polyphenols^(21)^. Sultanas are dried seedless green grapes and compared with Corinthian raisins are juicier and with lighter colour (golden-coloured raisins). Moreover, they are also less studied, in particular with regards to glycaemic responses^(20,22)^. It has been proposed that the combined intake of yogurt and dried fruit, including raisins, could provide probiotics, prebiotics, high-quality protein, important fatty acids, and a mixture of vitamins, minerals, and antioxidants that have the potential to exert synergistic effects on health^(23)^.

Taking the above into consideration, the aim of this study was to investigate the short-term effects of GMY containing the above-mentioned microbial strains as starters, Corinthian raisins (C), Sultana raisins (S), GMY combined with C (GMYC), containing equal amounts of available carbohydrates (25 g) on postprandial glycaemic responses, subjective satiety and BP.

## Materials and methods

### Subjects

A total of twelve participants (four men, eight women), between 18 and 50 years, were recruited by a variety of methods, including online advertisements and flyers and notices posted around the university campus were studied. Using the *t* distribution and assuming an average CV of within-individual variation of incremental AUC (iAUC) values of 25 %, *n* 10 participants has 80 % power to detect a 33 % difference in iAUC with two-tailed *P* < 0·05. In the current investigation, we enrolled and studied twelve participants. Participants underwent an initial screening and measurements included anthropometry (body weight, height, waist and hip circumference), fat percentage using double X-ray absorptiometry method (DXA, Lunar DPX Series, General Electric), blood pressure (Omron, Intellisense, HEM-907, Omron Hellas) and fasting blood glucose via finger prick (calibrated MediSmart Ruby glucose metres with a lancing device, Lilly-PHARMASERV SA). Additionally, a questionnaire on general health was completed. Subjects were non-smokers, had a healthy BMI (between 18·5 and 24·9 kg/m^2^), a normal BP (systolic pressure < 120 mmHg and a diastolic pressure < 80 mmHg), normal fasting blood glucose concentration via finger prick (< 100 mg/dl) and no medical conditions (i.e. diabetes mellitus, CVD, polycystic ovary syndrome, nephropathy, liver conditions, clinical depression and gastrointestinal disorders), were not pregnant or lactating, nor taking medications known to affect glycaemia and were not allergic or intolerant to the test foods. All twelve subjects completed all test food procedures and were included for analysis.

The study was conducted at the Laboratory of Dietetics and Quality of Life, Agricultural University of Athens, Greece. All participants gave their informed consent for inclusion before participating in the study. The study was conducted in accordance with the Declaration of Helsinki, and the protocol was approved by the Bioethics Committee of the Agricultural University of Athens (EIDE Reference Number: 24). This trial was registered at Clinicaltrials.gov (NCT05069506).

### Study design

**Fig. 1.**
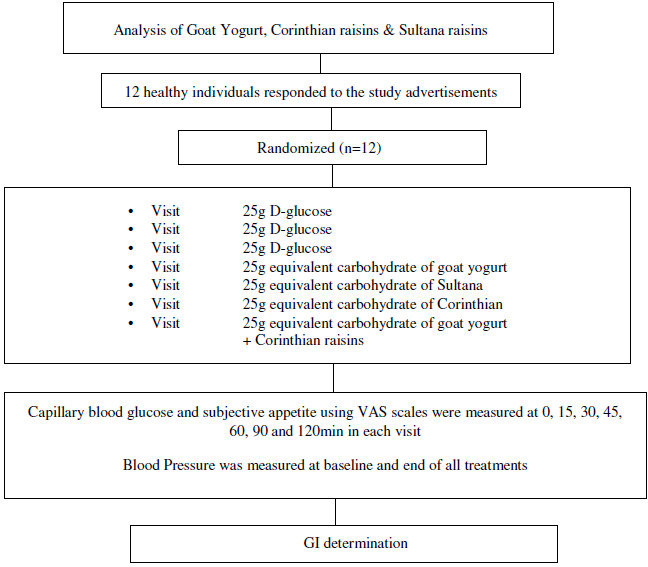
Schematic showing the outline of the study. Participants received, in a random order, the reference food (D-glucose), tested three times (i.e., 1^st^, 4^th^, 7^th^ visit), and the test foods goat milk yogurt (GMY), Sultana raisins (S), Corinthian raisins (C), and the combination of goat milk yogurt and C (GMYC), tested once, in different weeks, with a random sequence in accordance with the recommended glycemic index (GI) methodology. Abbreviations: VAS: visual analogue scales.

The GI of GMY, S, C and GMY in combination with C (GMYC) were evaluated. The GI was determined according to ISO 26 642: 2010 International Organization for Standardization^(24)^ methods and procedures (Figure 1). The study consisted of seven dietary treatments in a randomised, open-label type, cross-over design. Eligible participants were studied on four separate days over a period of 3–9 weeks with an interval of no less than 40 h and no more than 2 weeks between tests. Participants attended seven test sessions of around 3 h, separated by a wash-out period of at least 2 days. Online computer software (Social Psychology Network, Middletown, CT, USA) was used for simple randomisation of the sequence of the test foods (http://www.randomizer.org/) (accessed on January 2019)^(24)^. A researcher not involved in the collection and analysis of the scientific data was responsible for the randomisation of the volunteers to the intervention days examining the test foods. Participants arrived at the test centre around 08.45–09.00 h in the morning following an overnight fast of 10.00–14.00 h. Participants were asked to maintain stable dietary and activity habits throughout their participation in the study. In addition, participants were instructed to refrain from alcohol on the previous evening, from vigorous exercise on the morning of the test, and were only allowed to eat the provided foods throughout the test sessions. If any participant was not feeling well or had not complied with the preceding experimental conditions, the test was not carried out and was rescheduled for another day. On each test occasion, participants were weighed. Each session consisted of a test food that had to be consumed at a comfortable pace within 12–15 min and 2 h post-consumption measurement of metabolic blood parameters. Participants were instructed to consume the glucose drink at a comfortable pace within 10 min. During each of the seven test sessions, participants consumed one of the following test foods: GMY, S, C, GMYC, all tested once and glucose reference drink (25 g anhydrous D-glucose dissolved in 250 ml water) as reference food, tested three times in a nonconsecutive order (i.e. 1st, 4th and 7th visit), in different weeks, with a random sequence in accordance with the recommended GI methodology^(25,26)^ (Figure 1). All the test foods and the reference foods were given in portions containing 25 g available carbohydrates. The portion of 25 g available carbohydrates was chosen according to ISO 26 642: 2010 International Organization for Standardization^(25)^ because the portion size of GMY providing 50 g of glycaemic carbohydrate was unreasonably large (1·24 kg) to consume. Test meals were served with 300 mL water as a drink in all seven trials.

### Test foods

Food characteristics and macronutrient composition are shown in Table 1. The nutritional characteristics of the studied foods were evaluated in terms of their total protein content (Kjeldahl AACC 47–12), ash content (AOAC 923·03), moisture (AOAC 930·15), available carbohydrates and total dietary fibres (Megazyme kit-K-ACHDF, Megazyme Ltd., Scotland, UK), which calculates only the carbohydrates that can be absorbed (sugars and digestible starch), neglecting dietary fiber and resistant starch. Each portion of the tested foods (617·28 g GMY, 64·77 kcal; 37·48 g S, 312 kcal; 37·76 g C, 320 kcal; 308·64 g GMY and 18·88 g C, 193 kcal) or the reference food (25 g glucose, 95 kcal) was equivalent to a 25 g amount of available carbohydrates.

**Table 1.** Energy and macronutrient composition per serving containing 25 g available carbohydrates

	Corinthian raisins	Sultana raisins	Goat Milk Yogurt
Energy (kcal)	120·83	116·94	399·81
pH			4·60–4·90
Fat (g)	0·00	0·00	20·31
Carbohydrates (g)	27·56	26·99	25·00
Sugars (g)	27·19	26·61	25·00
Glucose (g)	18·46	18·31	0
Fructose (g)	18·51	18·37	0
Sucrose (g)	0·29	0·28	0
Maltose (g)	0·45	0·43	0
Lactose (g)	0	0	25
Protein (g)	1·10	1·12	29·26
Dietary fibre (g)	2·53	2·62	0
Amount of food for 25 g available carbohydrates	37·76	37·48	617·28

### Blood glucose concentrations

**Fig. 2.**
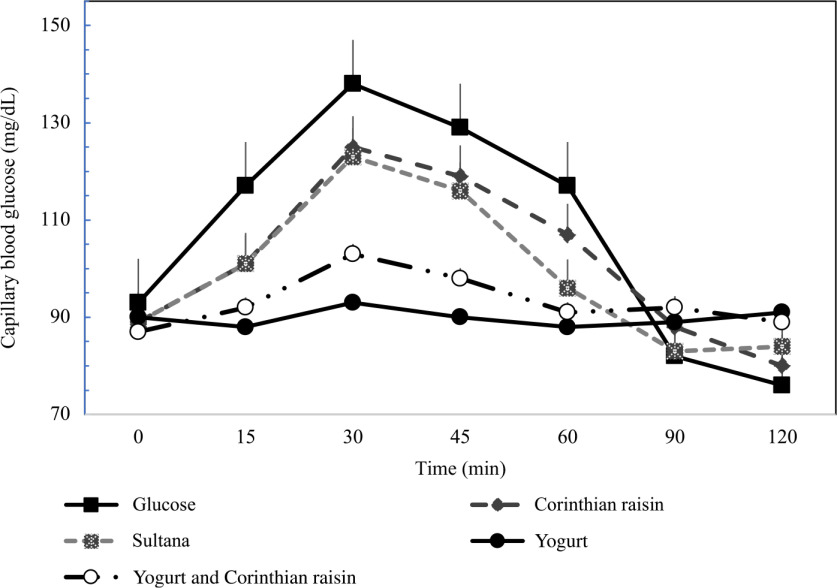
Postprandial glucose responses to four foods containing 25 g of available carbohydrates from D-glucose, goat milk yogurt (GMY), Sultana raisins (S), Corinthian raisins (C), and goat milk yogurt with Corinthian raisins (GMYC) (n=12). Values are means ± SEM.

To determine blood glucose concentrations, trained individuals from our research team performed the capillary blood glucose monitoring procedure by skin pricking according to the scheduled time. On each test occasion, three fasting blood samples were obtained by finger-stick at 5-min intervals (–10, −5 and 0); the average of the glucose concentrations at these three time points was taken to be the baseline (fasting) concentration. Then participants were served a test-food together with 300 ml water. They were instructed to consume all of the food and water at a comfortable pace within 12–15 min. Further finger-prick blood samples were collected at 15, 30, 45, 60, 90 and 120 min after starting to eat. Each blood glucose time value was the mean of three blood samples from the same drop of blood of each participant. Before and during the test, a blood glucose test record was filled out with the participant’s initials, identification number, date, body weight, test food, beverage, time of starting to eat, time it took to eat, time and composition of last meal and any unusual activities. During the 2 h test, participants remained seated quietly. After the last blood sample had been obtained, participants were offered a snack and then allowed to leave. To standardise all data collection procedures, capillary blood glucose monitoring was performed at the fingertip (distal phalange of the third finger). Blood glucose was measured with calibrated glucometers using glucose dehydrogenase-FAD test strips (Ruby Blood glucose Test Strips, Lilly-PHARMASELV S.A.), which show no reactivity to any sugars other than glucose and have better heat resistance and oxygen resistance. The repeatability and within laboratory coefficient variations were 3·2 %. The average blood glucose response curve was plotted by calculating the mean blood glucose concentrations of all participants at each time point (Fig. 2). Then for each sample and each study subject, the iAUC was calculated geometrically, using the trapezoid rule, and ignoring the area beneath the baseline^(25,27)^. The GI calculation for each test food sample used the method referred to as the mean of the ratios. For each subject, the ratio between the individual iAUC after consuming the test food sample and the iAUC for the same subject after consuming the reference food was calculated and expressed as a percentage value. Then, the GI of each test food was calculated as the average value of the ratios across all the subjects consuming the test food samples^(25,27)^. The mean, sd and coefficient of variation (CV = 100 × sd/mean) of the AUC of each subject’s repeated glucose (reference food) were calculated. The GL was calculated by multiplying the GI and the amount of available carbohydrate in the given amount of food and then dividing by 100 (GL = GI × available carbohydrates per serving/100)^(25,27)^. Peak blood glucose, defined as the highest recorded glucose value minus the baseline value, and peak blood glucose time, defined as the time elapsing from the start of a meal to the highest recorded glucose value, were calculated.

### Subjective satiety, blood pressure measurements and dietary intake analysis

Participants rated their hunger, desire to eat and perceived fullness after eating on 100 mm line visual analogue scales, ranging from not at all (0 mm) to extremely (100 mm), with for example neither hungry (0 mm), full (100 mm) or having desire for food in the middle (50 mm). Visual analogue scales were given in the form of a booklet, one scale per page^(28)^.

BP (systolic and diastolic) was measured at the beginning and the end of each test food intervention using an upper arm digital BP monitor (Omron, HEM-907, Omron Hellas) in a quiet, warm setting. Participants were rested for 5 min in the supine position with their arm supported at the level of the heart after which three BP measurements were taken by an already introduced member of our trained research team to avoid the ‘White Coat effect’, at 1 min intervals, with the three readings averaged.

Dietary intake was assessed by 24-h recalls at every visit and analysed using the Diet Analysis Plus program, as well as using Hellenic and European Food Composition Databases (http://www.eurifir.org.foodinformation/food-composition-databases-2/ (Accessed on April 2020). The databases were modified to include new foods and recipes. The purpose of collecting dietary intake was to confirm that participants refrained from changing their eating habits until the study was completed.

### Statistical analysis

Data distribution was tested using kernel density estimation (KDE) plots. Normally distributed continuous variables are presented as mean values ± standard error of the mean (sem), unless otherwise stated, and the skewed as median (first tertile and third tertile). Differences in baseline continuous variables were evaluated using ANOVA for normally distributed continuous variables, Kruskal–Wallis test for skewed continuous data and Pearson chi square test for categorical variables. Data were entered into a spreadsheet by two different individuals and the values compared with assure accurate transcription. Incremental areas under the glucose curves (AUC), ignoring area below fasting, were calculated. For the purposes of the AUC calculation, fasting glucose was taken to be mean of the first measurement of the blood glucose concentrations at times −10, −5 min and 0 min. The GI was calculated by expressing each participant’s AUC for the test food as a percentage of the same participant’s mean AUC for the three D-glucose drinks controls. If values were found to have > 2 sd above the mean, they would be excluded. No outlying GI values were found. The blood glucose concentrations at each time, AUC, GI values, subjective appetite and BP were subjected to repeated-measures ANOVA examining for the main effects of test food and the food x participant interaction. After demonstration of significant heterogeneity, the significance of the differences between individual means was assessed using Tukey’s test to adjust for multiple comparisons. Changes from baseline values were used in analyses when assessing the time and treatments effect for blood glucose differences and AUC measurements (blood glucose and subjective appetite). Absolute values were used to assess the treatment effect for variables measured from baseline to endpoints, such as BP. Means differing by more than the least significant difference were statistically significant, two-tailed *P* < 0·05. Glycaemic load (GL) was calculated using the formula: GL = GI x g of available carbohydrate in the portion. Data were analysed using SPSS 20·0 software (SPSS Inc.).

## Results

The subjects’ characteristics can be found in Table 2. There were no intermittent missing values or dropouts.

**Table 2.** Baseline participants’ characteristics (Mean values with their standard errors of the mean)

Characteristics		Total
*n*	12 (4 men, 8 women)	
Age (years)	23·50	0·70
Body weight (kg)	64·60	1·00
BMI (kg/m^2^)	22·67	0·89
Body fat (%)	35·90	2·50
Waist circumference (cm)	76·30	3·30
	Dietary intake (from 24-h recall)	
Energy intake (kcal)	1688·85	165·97
Protein (g)	67·02	7·66
Carbohydrates (g)	206·27	21·28
Fat (g)	65·17	7·51
Saturated fat (g)	21·58	2·71
Total cholesterol (mg)	218·74	26·99
Fibre (g)	18·06	1·96
Na (mg)	2648·74	509·83

Data are means ± sem.

### Glycaemic index and glycaemic load of test foods

The results of GI and GL for all tested meals are presented in Table 3. The results revealed that GMY was classified as low GI (GI ≤ 55 on glucose scale), C as high GI (GI ≥ 70 on glucose scale), S as high GI (GI (≥ 70 on glucose scale) and GMYC as low GI (GI ≤ 55 on glucose scale) food. Compared with the reference food (D-glucose), GMY and GMYC had significantly lower GI value (*P* < 0·05). All test foods were classified as low GL foods (GL ≤ 10 per serving; GMY: GL = 3; S: GL = 9; C: GL = 10; GMYC: GL: 6). Compared with the reference meal (D-glucose), all test foods had significantly lower GL value (*P*_for all_ < 0·05).

**Table 3.** Incremental area under the curve (iAUC) for blood glucose, glycaemic index (GI), glycaemic load (GL) and peak for blood glucose values of goat milk yogurt, Sultana raisins, Corinthian raisins and the combination of goat milk yogurt and Corinthian raisins food products, relative to the reference food D-glucose. (Mean values with their standard errors of the mean)

Food	iAUC (mmol 120 min/l^–1^)	GI	GL	Peak for blood glucose (mg/dL)
D-glucose (reference food)	2112·58 ± 188·30^a^	100	–	49·31 ± 3.60^a^
GMY	413·03 ± 181·84^b^	25.61 ± 7.12^c^	2.62 ± 0.71^ac^	8·29 ± 2·40^b^
S	1444·72 ± 254·28^ac^	69.68 ± 1.09^a^	9.30 ± 1.09^bc^	39·13 ± 5.99^a^
C	1638·54 ± 304·91^ac^	74.51 ± 10.18^a^	9.86 ± 1.35^b^	40·58 ± 4.43^a^
GMYC	946·08 ± 242·56^bc^	47.44 ± 9.44^bc^	5.54 ± 1.10^c^	18·21 ± 2.81^b^

Data are the means (± SEM). Each value represents the mean of twelve participants. Values marked with different superscript letter (a, b, c) are significantly different (*P* < 0·05). Means were compared column-wise by using one-way ANOVA for factor “treatment”, period and sequence of treatment, and post hoc Tukey test with Bonferroni correction to account for multiple comparisons between test meals; *P*-values < 0·05 were considered as significant. Abbreviations: GMY: goat milk yogurt; S: Sultana raisins; C: Corinthian raisins; GMYC: goat milk yogurt and Corinthian raisins.

### Blood glucose concentrations

Figure 2 describes the average blood glucose response curve showing the glucose responses of the test foods and reference food. No significant differences were observed on fasting glucose concentrations between glucose and the test foods (*p*_for all_ > 0·05). There was a significant blood glucose × time interaction (*P* = 0·004), a blood glucose × time × test food interaction (*P* < 0·001), a time × test food interaction (*P* < 0·001), a main effect of test food on blood glucose concentrations (*P* < 0·001), and a main effect of test food on GI and GL (*P*_for all_ < 0·001). Compared with the reference food (D-Glucose), lower blood glucose concentrations were observed as changes from baseline after the consumption of GMY at 15, 30, 45 and 60 min (*P*_for all_ < 0·001, respectively); for GMYC at 15, 30, 45 and 60 min (*P*_for all_ < 0·001, respectively); for C at 15 and 30 min (*P* < 0·001, *P* = 0·02, respectively) and for S at 15, 30 and 45 min (*P* < 0·001, *P* = 0·01 and *P* = 0·03, respectively). Compared with the reference food (D-glucose), higher glucose concentrations as changes from baseline at 120 min postprandially were observed for GMY, S and GMYC (*P* < 0·001, *P* = 0·04 and *P* = 0·001, respectively). Peak glucose rise was significantly lower for GMY and GMYC compared with the reference food (D-glucose), C and S (*P*_for all_ < 0·05), without differences between them (Table 3).

The 0–120 min iAUC for blood glucose values calculated for each test food are shown in Table 3. There was a significant main effect of test meal on iAUC for blood glucose (*P* < 0·001). The average intra-participant coefficient variation of iAUC values after the three repeated D-glucose tests was 30 %. The 0–120 min iAUC for blood glucose values calculated as changes from baseline for GMY and GMYC were significantly lower than those of the reference food (D-glucose), C and S (*P*_for all_ < 0·05; Table 3). No significant differences were observed as absolute values for time to peak for blood glucose.

### Subjective appetite and blood pressure

No significant differences were observed for subjective appetite ratings as changes from baseline and BP as absolute values at the end of all test food interventions.

## Discussion

By applying the standard GI methodology, this study produced data for GMY alone and the combination of GMY with Corinthian raisins, both being classified as low GI and low GL foods, attenuating the glycaemic responses, and significantly lowering peak blood glucose values. Both raisin varieties (Sultana and Corinthian) were classified as high GI and low GL foods.

The GI value difference observed based on the reference food has been thoroughly discussed^(29)^. The current investigation studied for the first time the GI of GMY that was found to be a low GI food, providing similarly low GI values as cow milk yogurts^(9)^. Two studies demonstrated that goat milk oral administration in STZ-induced rats with diabetes and in rats fed a high-fat diet improved glucose homoeostasis, promoted hepatic and skeletal muscle AMPK activation and increased fecal Lactobacillus abundance and propionic and butyric acids concentrations^(30,31)^. Another study in healthy humans showed that consumption of fermented goats’ milk for 3 weeks improved anti-atherogenicity in healthy subjects, prolonging resistance of the lipoprotein fraction to oxidation, lowering levels of peroxidised lipoproteins, oxidised LDL, 8-isoprostanes and glutathione redox ratio, enhancing total antioxidative activity and altering both the prevalence and proportion of lactic acid bacteria species in the gut microflora of the^(32)^. A recent review of the literature discussed several health benefits of goat milk and milk-based products including reduction of acute inflammation, lowering exaggerated basal secretion of IL-6, IL-8 and acute response and modest down-regulation of IL-1 beta and production of TMF-*α*in immune compromised elderly patients^(33)^. Moreover, it was discussed that goat milk and milk-based products improved cholesterol mobilisation and controlled its storage in the blood and had ACE inhibitory activity, particularly beta-lactoglobulin, possessing potential antihypertensive properties^(33)^.

The low GI value obtained for GMYC was also similar to other cow milk yogurt – fruit GI values (mean GI 41 ± 2, on glucose scale) and is in agreement with previous studies^(34)^. The current investigation demonstrated that the addition of Corinthian raisins to GMY, on the basis of isoenergetic exchange for other carbohydrates (displacing half of the available carbohydrate content from Corinthian raisins and half of the available carbohydrate content from GMY, keeping constant the available carbohydrate content at 25 g), resulted in significant reduction of GI and GL values and of glycaemic excursions, as well as lower peak glucose values, even when compared with both the reference food (D-glucose) and raisins consumed alone. Attenuating post-meal blood glucose excursions is a clinical challenge, as glucose fluctuations are known to induce oxidative stress and beta-cell damage^(35)^. Indeed, increased glucose variability from peaks to nadirs has been recognised as a major metabolic defect leading to CVD in people with type 2 diabetes^(36)^. Moreover, it has been shown that a low GI may be sufficient to achieve a lower glycaemic response from one meal to the next^(37–40)^.

On the other hand, it is recommended to the public to consume more fruit, together with vegetables and whole grains, as part of a healthy dietary approach in order to maintain health and prevent chronic disease development^(41)^. Whole fruits are typically low GI foods. The current investigation classified both raisin varieties (Sultanas and Corinthian) as high GI foods, which is in contrast to results from other studies, but as low GL foods which is in agreement with others^(20,42–48)^. The reason for this discrepancy may be the different variety of raisins used in studies originating from Canada, USA and Australia^(41,42,46,47)^, made from different grapes, containing about 11 g less sugars compared with the Greek varieties tested. Greek raisins contain predominantly fructose and glucose at almost equal amounts and low amounts of sucrose and maltose^(49)^. Another study from Greece examined the GI of Corinthian raisins and found it to be 66 ± 3^(20)^. However, in that study, the standard GI protocol was not followed, and GI values were provided only by estimation^(20)^. A flatter glycaemic response has been seen after consumption of whole fruit when compared with fruit puree and even more so when compared with drinking fruit juice^(50,51)^. However, in our study, a flatter glycaemic response was not observed with neither raisin variety. Moreover, although raisins are claimed to have a relatively high soluble dietary fiber content and high phytochemical content, all of which have been implicated in decreasing postprandial glucose concentrations^(52–55)^, no difference in peak glucose values were observed between Sultana and Corinthian raisins or when compared with the reference food.

Regarding satiety effects, it has been shown that goat milk products when consumed in place of cow milk products offer a slightly higher satiating effect, possibly due to increased glucagon like peptide secretion and decreased blood TAG levels^(56)^. One study showed that *ad libitum* consumption of raisins and grapes achieved low snack intake prior to dinner, compared with potato chips and cookies, in children aged 8–11 years^(57)^. Another study showed that the consumption of a premeal snack of raisins, but not grapes, or a mix of raisins and almonds, reduced meal-time energy intake and did not lead to increased cumulative energy intake in children^(58)^. The findings from the current investigation suggest that raisins may suppress appetite as much as the GMY treatment, which provides more than twice the energetic amount of the raisin treatments (> 3 times the glucose treatment) with higher amounts of protein and fats, both well known for their effects on satiety^(59–62)^.

In the current investigation, we failed to prove our GMY with peptides carrying the RPKHPINHQ fragment acute BP-lowering hypothesis. ACE-Inhibitory (ACE-I) peptides carrying the RPKHPINHQ fragment have been also identified in cow or sheep milk fermented with either *L. delbrueckii* subsp. *bulgaricus* ACA-DC 87 or *S. thermophilus* ACA-DC 835 strain^(18)^. This is rather expected, as RPKHPI(K/N)HQ is a highly conserved sequence of α_s1_ casein and conserved amino acids among the peptides containing this sequence can be responsible for their bioactivity. However, further studies should be performed to compare the ACE-I activity of yogurt produced from different types of milk. It has been reported that the mean arterial blood pressure as well as systolic and diastolic blood pressure are effectively lowered by ACE inhibitory pharmaceuticals both in hypertensive and normotensive people^(63,64)^. Few clinical trials in humans have shown that ACE-I tripeptides possess antihypertensive properties, but their effects are either inconclusive or pronounced only in people with established hypertension^(19,65,66)^. It is possible that the lack of effectiveness of GMY on BP could be possibly explained by the fact that there are two forms of the enzyme ACE in humans, namely somatic ACE which has two tandem active sites with distinct catalytic properties and sperm-specific germinal ACE, the function of which is largely unknown and has just a single active site, while an ACE homolog that differs from ACE, ACE2, has been also identified in humans^(67)^. These data highlight the possibility that the ACE-I peptides produced in GMY may not be able to target the individual active sites of all ACE enzymes and exhibit a BP-lowering effect in normotensive people. Additionally, the importance of the GMY matrix interactions with the ACE-I peptides should not be neglected, as the bioaccessibility and bioavailability of the peptides can be reduced and both the expected *in vitro* and/or *in vivo* biological activity may differ when the peptides interact with the complex mixture of components that are present in any food^(68,69)^. It may also be that one needs to consume GMY with peptides carrying the RPKHPINHQ fragment over a longer period of time for its BP-lowering effects to be manifested. In addition, more studies are needed to examine the effects of these ACE-I peptides on BP in people with and without established hypertension.

The strengths of our studies include the randomised, crossover design. Moreover, we tested for the first time the GI of GMY with ACE-I peptides and of a mixed meal made with GMY and Corinthian raisins. The major limitation of the present investigations, as with all acute feeding trials, is the inability to translate these acute findings to long-term benefits. Another shortcoming is the sample size. While the use of twelve participants has been validated by a number of studies, nevertheless this sample size reduces the study precision and may lead to exaggerated associations. Despite these limitations, this study adds to a growing body of evidence supporting that GMY and GMY with Corinthian raisins may be a dietary alternative to body weight and glycaemic control due to their low GI properties and flattening of the postprandial blood glucose response curve.

In conclusion, our results showed that GMY, C, S and GMYC foods differed in GI/GL. Based on our results, one may suggest that both GMY and GMYC are healthy dietary alternatives leading to attenuated postprandial glycaemic responses, which may offer advantages to glycaemic control not only for the general population but also for those with diabetes or impaired glucose tolerance.

## Supplementary material

For supplementary material accompanying this paper visit https://doi.org/10.1017/S0007114522002537. click here to view supplementary material
