# Supplementary material for: Short-term effects of goat milk yogurt-containing angiotensin-converting enzyme inhibitory peptides and two raisin varieties on subjective appetite, blood pressure and glycaemic responses in healthy adults. Results from a randomised clinical trial
Source: Br J Nutr. 2022 Aug 3;130(2):360–8. doi: 10.1017/S0007114522002537 (PMC10277659; doi:10.1017/S0007114522002537)
Supplement: Supplementary file 1 [file S0007114522002537sup001.docx]

**Supplementary material**

Yogurt preparation and analysis

Starters

*Lactobacillus delbrueckii* subsp. *bulgaricus* ACA-DC 87 and *Streptococcus thermophilus* ACA-DC 835 were used as starters in yogurt production. They were isolated from artisanal sheep and cow milk Greek yogurt, respectively, and belong to the ACA-DC Collection of Microorganisms (Laboratory of Dairy Research, Agricultural University of Athens, Greece).

For the preparation of the yogurt production inoculum, strains were first subcultured (inoculum 1%, v/v) overnight in reconstituted skim milk powder (10%, w/w), enriched with yeast extract (0.3%, w/w), and subsequently in the goat milk (inoculum 1%, v/v), which was used for yogurt production, in both cases overnight at 37 ^o^C under microaerophilic conditions.

Yogurt production

Commercial goat milk (fat 3.5 %, w/w; protein 3.6 %, w/w; lactose 4.4 %, w/w; total solids 11.58 %, w/w; pH 6,7) was heat treated at 95 ^o^C for 10 min and then cooled down to 4 ^o^C. *L. bulgaricus* ACA-DC 87 and *S. thermophilus* ACA-DC 835, subcultured overnight in goat milk (37 ^o^C), were used as inoculum for yogurt production (total inoculum 4 % v/v, in a 1:1 ratio). The batch was then poured into sterilized glass screw jars (100 mL) and incubated at 42 ^o^C until a pH of 4.90 was reached. After 30 min of tempering, yogurt was transferred to a cold room (4 ^o^C) and stored for 24 h before administration. Additional yogurt samples were stored for 28 days and microbiologically analyzed in one-week intervals.

## Physicochemical analysis of yogurt

The pH of yogurt samples stored at 4 ^o^C for up to 28 days was recorded weekly using a 632 pH Meter (Metrohm Herisau, Switzerland). Fat, total nitrogen-protein, lactose and total solids were determined in the yogurt used for the clinical trial. Fat was determined by the ISO 2446: 2008 (1). Total protein content was performed in accordance with ISO 8968-1: 2014 (2). Lactose was determined in accordance with the standard method ISO 22662: 2007 which provides High Performance Liquid Chromatography (HPLC) (3). Total solids, reflecting the moisture content of yogurt, were determined by drying samples overnight at 110 ^o^C to constant weight according to IDF 4A (4). Finally, determination and expression of the yogurt ACE-I activity were performed as described by Georgalaki et al. (18).

Enumeration of *L. bulgaricus* ACA-DC 87, *S. thermophilus* ACA-DC 835 and spoilage microorganisms

All microorganisms were enumerated in yogurt samples stored at 4 ^o^C on days 0, 1, 7, 14, 21 and 28. Analysis was performed in triplicate. Yogurt (10 g) was diluted with 90 mL sterilized Ringer solution, homogenized in a stomacher (Stomacher 400 circulator, Seward Limited, Norfolk, UK) and subsequent decimal dilutions in Ringer solution were performed in duplicate.

Viable counts of *L*. *bulgaricus* ACA-DC 87 were determined on MRS agar (pH 5.4; Oxoid, Basingstoke, Hampshire, UK) under anaerobic conditions (double agar layer) after incubation at 37 °C for 48-72 h, while viable counts of *S. thermophilus* ACA-DC 835 were enumerated on Milk Plate Count agar (Oxoid), after microaerophilic incubation at 37 °C for 48 h.

Moreover, all samples were examined for the presence of spoilage microorganisms, namely coliforms on VRBL agar (Biokar Diagnostics, Beauvais, France) at 37 ^o^C for 24 h, and yeasts and molds on YGC agar (Merck, Darmstadt, Germany) at 25 ^o^C for 5 days.

In all cases, plates containing 10 to 300 colony-forming units (CFU) were counted and the counts were expressed as log CFU g ^-1^ of yogurt.

Yogurt sensory analysis

Yogurt administered to the volunteers of the clinical trials was sensorially evaluated by a 45-member non-trained tasting panel, comprising of students from the Agricultural University of Athens. The panel rated the following attributes: (i) texture (consistency, viscosity, rough or sticky sensation on the tongue surface), (ii) flavor (goat smell, fermented milk like, lactic acid like, fruit or cheese perception) and (iii) taste (lactic acid, sweet, bitter, salty), using a 5-point product specific scale anchored on the left with «dislike» and on the right with «like» as follows 1: dislike very much; 2: dislike moderately; 3: neither dislike nor like; 4: like moderately; and 5: like very much (5).

Microbiological analysis of raisins

Two Greek raisins varieties, namely, the Corinthian, a protected designation of origin (PDO) product, and the Sultana, were used in the present study. Samples (10 g) were mixed with 90 mL of sterile peptone water and the suspension was left for 30 min at room temperature so as natural epiphytes be dissolved (6). Then, they were homogenized (Stomacher 400 circulator), and serial decimal dilutions were prepared in sterilized ringer solution and poured or plated in the appropriate agar media for the enumeration of the following groups: (i) Total bacteria count (PCA, Oxoid, 30 °C, 72 h); (ii) lactobacilli (MRS agar pH 5.4, Oxoid; 42 °C, 48 h, anaerobically using the gas pack plus anaerobic system (BBL, Becton Dickinson, Sparks, Maryland, USA), (iii) thermophilic cocci (M17 agar, Oxoid; 37°C, 48 h; (iv) enterococci on KAA (Oxoid); at 37 ℃ for 24 h; (v) micrococci on mannitol salt agar (Oxoid) supplemented with cycloheximide (100 mg/ml; Sigma-Aldrich St. Louis, USA) at 30 ℃ for 72 h; (vi) coliforms on violet red bile agar (Biokar, Beauvais, France) at 30 ℃ for 24 h (and (vii) yeasts/moulds on yeast glucose chloramphenicol (YGC) plates (Merc, Darmstadt, Germany) at 30 ℃ for 72 h.

Regarding the presence of presumptive *Salmonella*, 25 grams of raisins were suspended in 225 ml of buffered peptone water (pH 7.2; Sigma-Aldrich) and incubated for 24 h at 37 °C for pre-enrichment. One milliliter of the pre-enriched broth was transferred to 10 ml of selective enrichment tetrathionate broth (Oxoid) and incubated at 42 ^o^C. One loop of the overnight culture was streaked on the selective Salmonella Shigella agar SS (Oxoid) and incubated at 37 ^o^C under microaerophilic conditions.

**Results**

Yogurt

Physicochemical analysis of yogurt

The yogurt administered to the subjects of the clinical study had pH 4.6, while fat, protein, lactose and total solids content were determined 3.29, 3.70, 4.05 and 12.65 (%, w/w), respectively. The pH value is in accordance with existing literature data on yogurt produced from goat milk (7).

The % ACE-I activity was measured in GMY and was found to be 47.38 %, confirming the production of bioactive peptides by the yogurt starters leading to inhibition of the ACE enzyme.

Microbiological analysis of yogurt

Counts of *L. bulgaricus* ACA-DC 87 and *S. thermophilus* ACA-DC 835 are shown in Table 3. *L. bulgaricus* ACA-DC 87 and *S. thermophilus* ACA-DC 835 were used as starters (total inoculum 4 % v/v, in a 1:1 ratio), with initial counts of 7.23 and 5.04 log CFU mL^-1^, respectively. In the yogurt of day 1, which was administered to the subjects of the clinical study, counts reached 8.78 and 7.33 log CFU g^-1^ for *L. bulgaricus* ACA-DC 87 and *S. thermophilus* ACA-DC 835, respectively. These counts are above the minimum requirement of 7 log CFU g^-1^ of viable microorganisms, according to the Code of Food and Beverages (GFC, 1987; replacement of article 82: yoghurt) and WΗΟ/FAO (2003).

During yogurt storage at 4 ^o^C, counts of *L. bulgaricus* ACA-DC 87 reached a maximum value on day 14 (9.25 log CFU g^-1^) and then declined to 6.32 log CFU g^-1^ (day 28), while counts of *S. thermophilus* ACA-DC 835, after day 1 gradually declined to 5 log CFU g^-1^ (day 28) (Table 3). These results are in contrast to those of previous studies (8-10), which report that *S. thermophilus* retained high viable counts during the cold storage period. This might be attributed to the antagonism and competition between the two specific strains. No other microorganisms were detected in any of the yogurt samples, so yogurt was considered as safe.

**Table 1.** Counts of *L. bulgaricus* ACA-DC 87 and *S. thermophilus* ACA-DC 835, and pH during yogurt storage at 4°C

| **Time (days)** | **pH** | ***L. bulgaricus* ACA-DC 87 (log CFU mL^-1^)** | ***S. thermophilus* ACA-DC 835 (log CFU mL^-1^)** |
| --- | --- | --- | --- |
| 0 | 4.90±0.2 | 7.23±0.18 | 5.04±0.23 |
| 1 | 4.60±0.1 | 8.78±0.15 | 7.33±0.20 |
| 7 | 4.35±0.25 | 9.00±0.16 | 6.57±0.18 |
| 14 | 4.30±0.18 | 9.25±0.10 | 6.45±0.17 |
| 21 | 4.30±0.20 | 7.00±0.22 | 6.00±0.22 |
| 28 | 4.30±0.15 | 6.32±0.25 | 5.00±0.25 |

Sensory evaluation

Results of the sensory evaluation of yogurt administered to the subjects are shown in Figure 2. In general, texture, taste and appearance scored 4 (i.e., like moderately) by 35.56% of the panelists. Regarding texture, 57.78% of the panelists characterized yogurt as a product of reduced consistency and increased wateriness, while 60 % did not sense any bitter taste. Flavor, scoring as a whole 3 (i.e., neither dislike nor like), was characterized as goat smell, fermented milk like and lactic acid like, by 28.89, 40.00 and 33.00% of the panelists, respectively, while 38.00 and 29.00% detected fruity and cheesy flavor, respectively.

Several studies have shown that goat milk affects the sensory characteristics of fermented milks or yogurt, regarding firmness, consistency, syneresis and viscosity (11-13). On the other hand, goat milk yogurt scored significantly higher than yogurt prepared from cow and sheep milk for specific attributes at the level of taste (fatty, rich, delicious and whey), aftertaste (persistent, intense, goaty) and texture (velvety, smooth, fluid) (14). Moreover, according to (15), sensory analysis panelists indicated that yogurt prepared from mixtures of cow and goat milk received high scores regarding color, acidity and flavor, even at a 50 % goat milk content. This is also in accordance with recent studies, which suggest that the use of mixtures of different types of milk has a high potential in industrial production of functional dairy products (16, 17).

**Figure 1.**

**Figure 1 Legend: Spiderweb plot of yogurt sensory analysis**

**References**

1. Kleyn DH, Lynch JM, Barbano DM, Bloom MJ, Mitchell MW. Determination of fat in raw and processed milks by the Gerber method: collaborative study. J AOAC Int. 2001;84(5):1499-508.

2. Federation IID. 20B Milk. Determination of nitrogen content. Part 1: Kjeldahl method. International Dairy Federation, Brussels, Belgium. 1993.

3. Sarantinopoulos P, Kalantzopoulos G, Tsakalidou E. Effect of Enterococcus faecium on microbiological, physicochemical and sensory characteristics of Greek Feta cheese. Int J Food Microbiol. 2002;76(1-2):93-105.

4. Federation IID. Standard 4A: Determination of the Total Solids Content in Cheese and Processed Cheese. Brussels: International Dairy Federation. 1982.

5. Meilgaard MMGV, Civille GV, Carr BT. Selection and training of panel members. . Sensory Evaluation Techniques. 1999;3:174-6.

6. Bosnea LA, Kopsahelis N, Kokkali V, Terpou A, Kanellaki M. Production of a novel probiotic yogurt by incorporation of L. casei enriched fresh apple pieces, dried raisins and wheat grains. . Food and Bioproducts Processing. 2017;102:62-71.

7. Moschopoulou E, Sakkas L, Zoidou E, Theodorou G, Sgouridou E, Kalathaki C, et al. Effect of milk kind and storage on the biochemical, textural and biofunctional characteristics of set-type yoghurt. . International Dairy Journal. 2018;77:47-55.

8. Dimitrellou D, Salamoura C, Kontogianni A, Katsipi D, Kandylis P, Zakynthinos G, et al. Effect of Milk Type on the Microbiological, Physicochemical and Sensory Characteristics of Probiotic Fermented Milk. Microorganisms. 2019;7(9).

9. Lopes RP, Mota MJ, Pinto CA, Sousa S, da Silva JAL, Gomes AM, et al. Physicochemical and microbial changes in yogurts produced under different pressure and temperature conditions. LWT - Food Science and Technology. 2019;99:423-30.

10. Zoidou E, Theodorou S, Moschopoulou E, Sakkas L, Theodorou G, Chatzigeorgiou A, et al. Set-style yoghurts made from goat milk bases fortified with whey protein concentrates. . Journal of Dairy Research. 2019;86(3):1-7.

11. Vargas M, Cháfer M, Albors A, Chiralt A, González-Martínez C. Physicochemical and sensory characteristics of yoghurt produced from mixtures of cows' and goats' milk. . International Dairy Journal. 2008;18(2):1146-52.

12. Domagała J. Instrumental texture, syneresis and microstructure of yoghurts prepared from goat, cow and sheep milk. International Journal of Food Properties. 2009;12(3):605-15.

13. Miocinovic J, Miloradovic Z, Josipovic M, Nedeljkovic A, Radovanovic M, Pudja P. Rheological and textural properties of goat and cow milk set type yoghurts. . International Dairy Journal. 2016;58:43-5.

14. Megalemou K, Sioriki E, Lordan R, Dermiki M, Nasopoulou C, Zabetakis I. Evaluation of sensory and in vitro anti-thrombotic properties of traditional Greek yogurts derived from different types of milk. Heliyon. 2017;3(1):e00227.

15. Costa RG, Beltrão Filho EM, de Sousa S, da Cruz GRB, Queiroga RDCRDE, da Cruz EN. Physicochemical and sensory characteristics of yoghurts made from goat and cow milk. . Animal Science Journal. 2016;87(5):703-9.

16. Ranadheera CS, Evans CA, Adams M, Baines SK. Co-culturing of probiotics influences the microbial and physico-chemical properties but not sensory quality of fermented dairy drink made from goats’ milk. . Small Ruminant Research. 2016;136:104-8.

17. Champagne CP, da Cruz AG, Daga M. Strategies to improve the functionality of probiotics in supplements and foods. Current Opinion in Food Science. 2018;22:160-6.
